# Research on land‐use evolution and ecosystem services value response in mountainous counties based on the SD‐PLUS model

**DOI:** 10.1002/ece3.9431

**Published:** 2022-10-27

**Authors:** Yao Li, Jiulin Li, Jinlong Chu

**Affiliations:** ^1^ School of Social Development and Public Policy Beijing Normal University Beijing China; ^2^ School of Architecture and Urban Planning Anhui Jianzhu University Hefei China

**Keywords:** driving factors, ecosystem services, land‐use change, mountain areas, the PLUS model, the SD model

## Abstract

Rapid urbanization has caused changes in climate and environment and threatened the ecosystem with multiple risks. The ecosystem services capacity has shown a downward trend accordingly. It is significant to explore the spatio‐temporal evolution of land use and ecosystem services value (ESV) in mountainous counties at small scales, as it coordinates economic growth and ecological protection, and promotes sustainable and high‐quality development. Based on the SD‐PLUS model, the study simulated three scenarios of land‐use change in Qianshan city from 2019 to 2035: high‐growth rate, medium‐growth rate, and low‐growth rate, and studied the impacts of land‐use change on the ESV. Results showed that: (1) Under the three scenarios, the construction land in the study area increased significantly, the forest and water have a decreasing trend, and the scale of gardens partly increased. (2) In the urban built‐up areas, a significant amount of construction land is centrally expanded, whereas, in mountainous regions, construction land exhibits sporadic point expansion. And among the various factors that influence land‐use change, the impact of roads at all levels is the most significant, followed by elevation. (3) The overall ESV shows a downward trend, with the low‐growth rate scenario dropping the least (4.91%). The value distribution changes little at the space scale, and different regions demonstrate different degrees of change. From the perspective of value type, the service values of water conservation and waste treatment are significantly reduced, while that of food production is relatively stable; from the perspective of various lands with their ESV, cultivated land and forest remain stable. The study results can provide technical ideas for the coordinated economic development and ecological protection of mountainous cities and boost the implementation of green development.

## Graphical abstract

Based on the SD‐PLUS model, taking Qianshan city as an example, the study simulated three scenarios of land use change: ecological protection, coordinated development, and economic priority, and studied the impacts of land use change on the value of ecosystem services. Under the three scenarios, the construction land in the study area increased significantly, the forest and water area have a decreasing trend, and the scale of gardens has partly increased. Construction land expands in clusters in the urban built‐up areas and dots in mountainous areas; land use changes are primarily affected by roads, followed by areas where artificial facilities are relatively sparse, and DEM has the greatest impact on land use changes. The overall ecosystem service value shows a downward trend, with the comprehensive coordination type dropping the least.

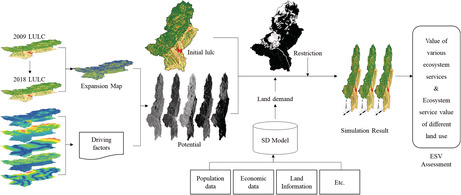

## 1 INTRODUCTION

Biodiversity and ecosystem services (ESs) not only provide people with necessary living goods and means of production but also have multiple ecological, economic, and social values, which are the material basis for human survival (Myers et al., 2000), and a fundamental guarantee for social stability and sustainable development (Tittensor et al., 2014). The degradation of ecosystem functions and the reduction of biodiversity is one of the major ecological and environmental crises facing the world today (Butchart et al., 2010). The proposal of ESs provides a new perspective and basis for biodiversity conservation (Coates, 2018; Silvis, 2012). ESs refer to the natural environment and utility of the ecosystem through its structure and function to sustain human existence (Peh et al., 2014). These services include regulating services, provisioning services, habitat services, etc (de Groot et al., 2012). Ecosystem services value (ESV) is the quantification of the value of natural resources or ESs from a natural ecological perspective, using ecological and economic research methods (Costanza et al., 1997). Land‐use change affects the function and structure of ESs by changing the land cover (Song et al., 2018), resulting in changes in ecosystem types, gradual degradation of ESs, and a decline in biodiversity (Larsen, 2004; Schneider et al., 2020), which leads to changes in ESV (Bryan et al., 2018; Kesgin Atak & Ersoy Tonyaloğlu, 2020). Optimizing the land‐use pattern according to ESV changes and comprehensively enhancing ESs are of great significance to ecosystem and biodiversity conservation.

Since China's reform and opening up, the rapid and large‐scale urbanization has caused drastic changes in land use (Hou & Wen, 2020), which is also an important reason for the changes in terrestrial ecosystems and the loss of biodiversity and ESs (Haines‐Young, 2009; Wang et al., 2019). Current research focuses on analyzing the spatio‐temporal changes in land use and the response of ESV. However, the majority put their emphasis on land‐use change and ESVs of big cities (Dadashpoor & Panahi, 2021), ecologically sensitive areas, and developed coastal areas (Liu et al., 2019). Research on small‐scale counties, especially mountain areas, remains inadequate. Mountain areas are crucial for the survival and sustainability of many human societies (Perrigo et al., 2020; Rahbek et al., 2019). On the one hand, mountains are known as cradles of diversity because they provide habitat and refugia for many species (Payne et al., 2020). And mountain ecosystems provide a vast array of resources (such as trees, food, clean air, or arable land) that are essential to subsistence activities, both for people living in the mountains and for those living outside the mountains (Liu et al., 2022). On the other hand, the mountain areas also undertake the function of ecological conservation to protect mankind from the impact of natural disasters (Crouzat et al., 2015; Li et al., 2022) and support urban development (Martín‐López et al., 2019). As important actors in regional strategy and policy, county‐level regions have played an increasingly significant role in the development of China's social economy (Yang et al., 2019). Therefore, refined simulation of land‐use changes and quantitative assessment of ESV in mountainous areas at the county scale could help coordinate economic development and ecological protection in the area, as well as improve the efficiency of land use, to assist policymakers in making more scientifically based decisions regarding strategies and promoting regional sustainable development.

Spatial‐temporal LULC (land use and land cover) models, which can analyze the complex structure of links and feedback by simulating future land‐use trajectories, are useful and easy‐to‐use tools for figuring out the causes and effects of possible future land‐use patterns concerning socio‐economic and natural environmental factors (Costanza & Ruth, 1998). Cellular automata (CA) models, as a “bottom‐up” spatial representation of dynamic systems, are prevalent approaches for simulating the spatial development of LULC by predicting the state of a cell based on its starting state, neighborhood impacts, and a set of transition rules (Liu, Liang, et al., 2017; Liu, Zheng, et al., 2017). An increasing amount of research has detailed the applicability of CA models in urban development studies during the last two decades. Nevertheless, the majority of CA models can only simulate the dynamics of single land use (Liu, Liang, et al., 2017; Liu, Zheng, et al., 2017), and they cannot easily address how macro‐scale regulation, economic development, and population migration influence micro‐units (Arowolo & Deng, 2018). Furthermore, in CA models, all cells follow the same transition rules; special cells are not recognized (Liu, Liang, et al., 2017; Liu, Zheng, et al., 2017).

Compared with the CA model, the SD (system dynamics) model is a top‐down macroscopic simulation of land use (Coyle, 1997). Using SD model, it is possible to simulate complex interactions between components under multiple “hypothesis” situations and to depict the flow and feedback linkages between distinct characteristics (Costanza & Ruth, 1998; Haghani Sang Lee Joon H Byun et al., 2003). However, the SD model cannot simulate individual behavior (Liu, Liang, et al., 2017; Liu, Zheng, et al., 2017), and lacks the ability to manage and analyze geospatial data (Guo et al., 2001).

SD models can more accurately replicate the geographical distribution of land use when paired with CA models (Liang et al., 2018). Researchers have created many land‐use simulation models (Liu, Liang, et al., 2017; Liu, Zheng, et al., 2017), the most popular of which are Logistic‐CA (Nasiri et al., 2019), CA‐Markov model (Rahnama, 2021), ANN‐CA (Ullah et al., 2019), CLUE‐S (Clerici et al., 2019), Fore‐SCE (Liang et al., 2020), and FLUS (Liu, Liang, et al., 2017; Liu, Zheng, et al., 2017). However, these models are ineffective at identifying the variables influencing LULC, do not allow modeling of different land‐use patches, particularly natural land‐use types, in a dynamic spatio‐temporal way, and cannot obtain land‐use change rules at certain time intervals (Wang et al., 2022). The recently developed advanced patch‐generating land‐use simulation (PLUS) model can determine the development potential of each land‐use type via the Random Forest (RF) algorithm, allowing for more accurate simulations of changes in the spatial distribution of land use (Liang et al., 2021).

Thus, it is urgently needed to study the response of ESV to land use in small‐scale mountain areas. To this end, using Qianshan city in southeast China as an example, the objectives of this study are as follows: (a) to simulate land‐use changes under multiple scenarios; (b) to explore the potential causes of land‐use evolution; (c) to assess ESV and analyze temporal‐spatial ESV changes. In summary, this study, by augmenting previous research on the prediction and modeling of land‐use patterns, is hoped to provide scientific support for multi‐scale territorial and spatial planning and regional sustainable development.

## 2 RESEARCH AREA AND DATA SOURCE

### 2.1 Overview of the study area

**FIGURE 1:**
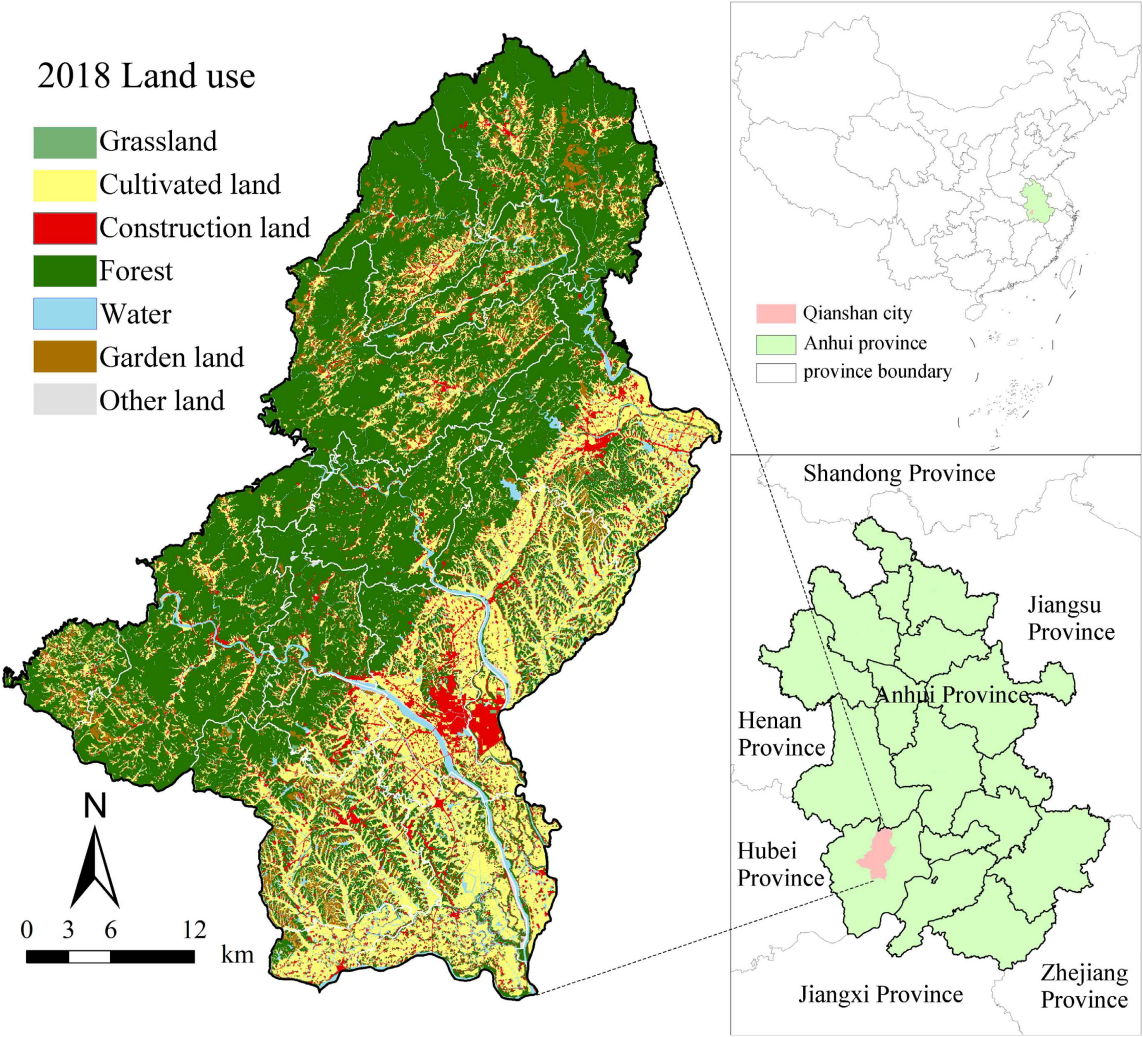
Spatial location of Qianshan in China

Qianshan City (30°27′–31°04′N; 116°14′–116°46′E) is located in southwest Anhui (Figure 1), located in the subtropical monsoon climate zone. It has a total area of 1688 km^2^, of which 49.3% is made up of mountains and 9.9% is made up of hills. According to the Qianshan Forestry Bureau, by the end of 2021, the forest covers 940 km^2^ of land in Qianshan City, which has a total volume of 4.91 × 10^6^ m^3^ and is mostly broad‐leaved deciduous forests. Qianshan City is rich in plant and animal resources, with 1857 kinds of forest plant resources such as ginkgo, cedar, horsetail pine, etc. There are 127 kinds of wild animals, such as otters and small ling cats, among which there are 26 kinds of rare animals. Since the beginning of the 21st century, urbanization has accelerated in Qianshan, and the area of construction has rapidly expanded. This has led to changes in ecosystem structure such as water pollution, forest degradation, and soil erosion, and has threatened the stability of the ecological environment (Miao et al., 2016). At present, Qianshan has been listed as an extremely important region for ecological protection by the Ministry of Ecology and Environment of the People's Republic of China. However, the ecosystem is still under tremendous pressure.

### 2.2 Data source and processing

The socio‐economic data of Qianshan city used in the study comes from the “Statistical Yearbook of China Urban Construction,” “Statistical Yearbook of Anhui Province,” “Statistical Yearbook of Anqing City,” and “Statistical Yearbook of Qianshan city” and statistical information of land‐use data. Statistical indicators include GDP (Gross Domestic Product), total population, fixed asset investment, urban and rural population, population change rate, GDP change rate, food production, etc.

The data on land use, administrative boundaries, and road network information over the years are from Qianshan Natural Resources Bureau. The elevation data are obtained from the Geospatial Data Cloud platform (http://www.gscloud.cn/). In addition, the points of interest in the study area, such as government agencies, shops, and supermarkets, are crawled in the Baidu Map open platform (https://lbsyun.baidu.com/). The population spatial distribution data comes from the WorldPop open spatial demographic data and research (https://www.worldpop.org/geodata/listing?id=69), with an accuracy of 100 m. The night light data are obtained from the Wuhan University LJ‐1 Night Light Remote Sensing Data set in the High‐resolution Earth observation system Hubei Data and Application Center (http://59.175.109.173:8888), with a spatial resolution of 130 m. All spatial data uniformly use the WGS_1984_UTM_Zone_50N coordinate system and resample to 10m spatial resolution.

## 3 RESEARCH METHOD

**FIGURE 2:**
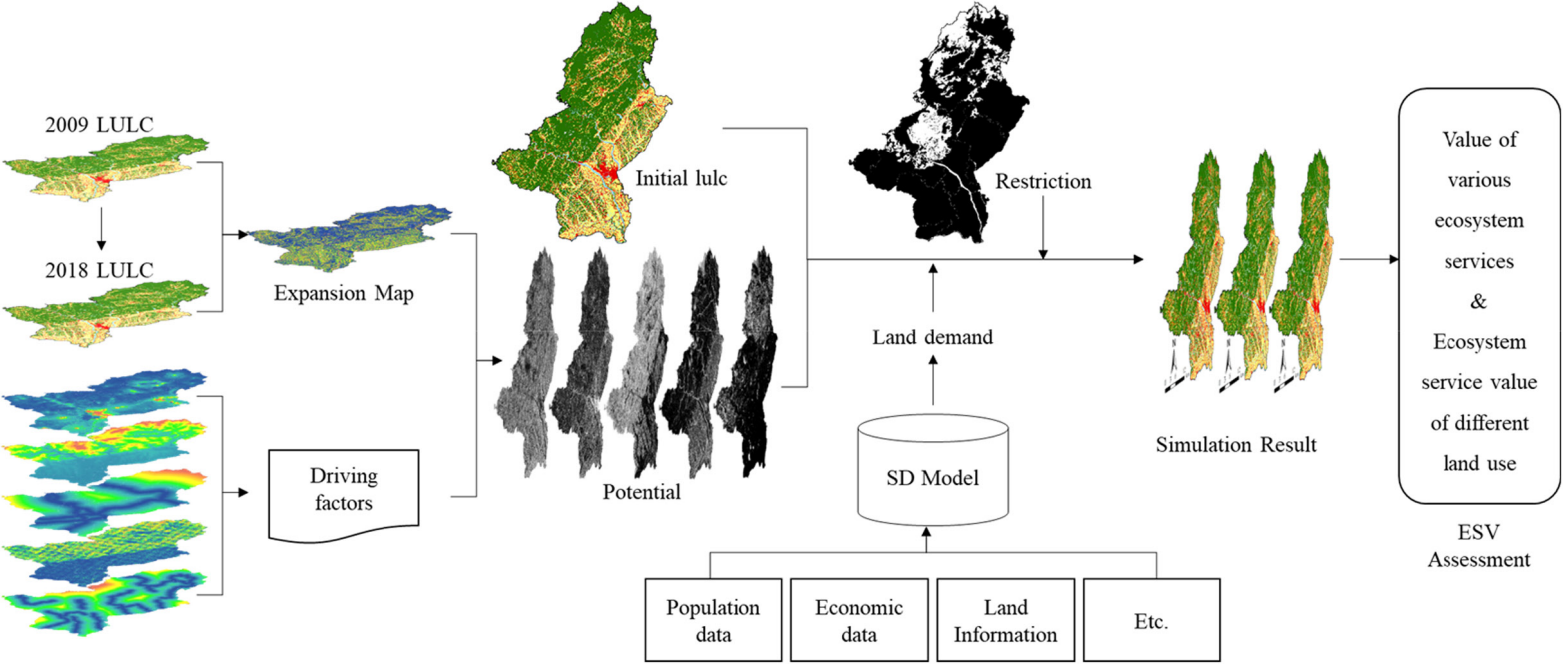
Flow chart of SD‐PLUS model. (LULC, Land use and land cover; ESV, ecosystem services values; Potential, potential map; Restriction, restriction map)

The study contains three sub‐models. First, the system dynamics model is used to predict the demand for land‐use change under the influence of various social and economic factors. Second, the PLUS model is used to simulate land‐use change on land demand under multiple scenarios. At last, the ESV assessment model is used to explore the ESV response toward land‐use changes under different scenarios (Figure 2).

### 3.1 System dynamics model

#### 3.1.1 Model construction

The SD model regards the study area as a relatively independent system. The increase or decrease of the total population affects the change in people's demand for different land types, and the variables selected in the study mainly include the total population, urban population, and population change rate. Urban construction land has expanded rapidly in the process of urbanization. At the same time, the phenomenon of "one family with many houses" is widespread in rural areas, therefore, rural construction land is also increasing.

With the rapid development of the economy, investment in fixed assets has increased year‐by‐year, and a large amount of funds has been invested in land development such as transportation and commercial services, further promoting the expansion of construction land. On the other hand, the growth of economic strength has also played an important supporting role in the restoration of cultivated land, conservation of forest and water, and other natural resources. From the perspective of the transformation of various types of land, the expansion of construction land is inhibited to a certain extent. The indicators selected in the economic aspect mainly include GDP, GDP increase, investment in fixed assets, and so on.

The changes in various types of land use are the results of the SD simulation, mainly including construction land, forest, cultivated land, water, etc. Stimulated by population and economic development, the expansion of construction land will occupy a large amount of cultivated land, forest, and other land with good basic conditions.

According to the causality between the elements, a variable set of the SD model has been built up in the study. Moreover, existing literature (Gu et al., 2017; Liu et al., 2020; Tan et al., 2019) and scholars in related fields suggest that the SD model is a certain degree of simplification of reality, and cannot fully depict the real situation of reality, so the scope of the study can only be defined by determining the system boundary. This study's SD model focuses primarily on the alteration of land use in mountainous regions from 2018 to 2035, and its spatial scope is the administrative division boundary of Qianshan City. In addition, this study examines the development of the general situation, as the extreme case is not considered. As described below in the SD model parameter setting, the 2011 extreme value will be omitted, as it is not in line with the general trend in most years. Meanwhile, to simplify the calculation, we suppose that the land‐use change is affected only by human activities and ignores natural variation. Considering that the change in construction land is most severely affected by population and economy, the area of construction land and other types of land is obtained by simulating demographic and economic changes in the model (Equation 1).

(1) $${\text{SCON}}_{t}=\text{SCONt0}-3.35e^{-5*}\;{\text{URPOP}}_{t}+5.72{\text{7e}}^{-6*}\;{\text{INVEST}}_{t}$$

(2) $${\mathrm{GDP}}_{t}=\text{INTEG}\;\left({\mathrm{GDP}}_{\text{change}}\right)$$

(3) $${\text{URPOP}}_{t}=\text{INTEG}\;\left({\mathrm{POP}}_{\text{change}}\right)$$

where SCON_*t*_ indicates the construction land area in *t* year, SCON_*t*0_ is the construction land area in the base year, URPOP_*t*_ is the urban population size in *t* year, INVEST_*t*_ is the fixed asset investment scale in *t* year, and GDP_*t*_ is the GDP scale in *t* year.

**FIGURE 3:**
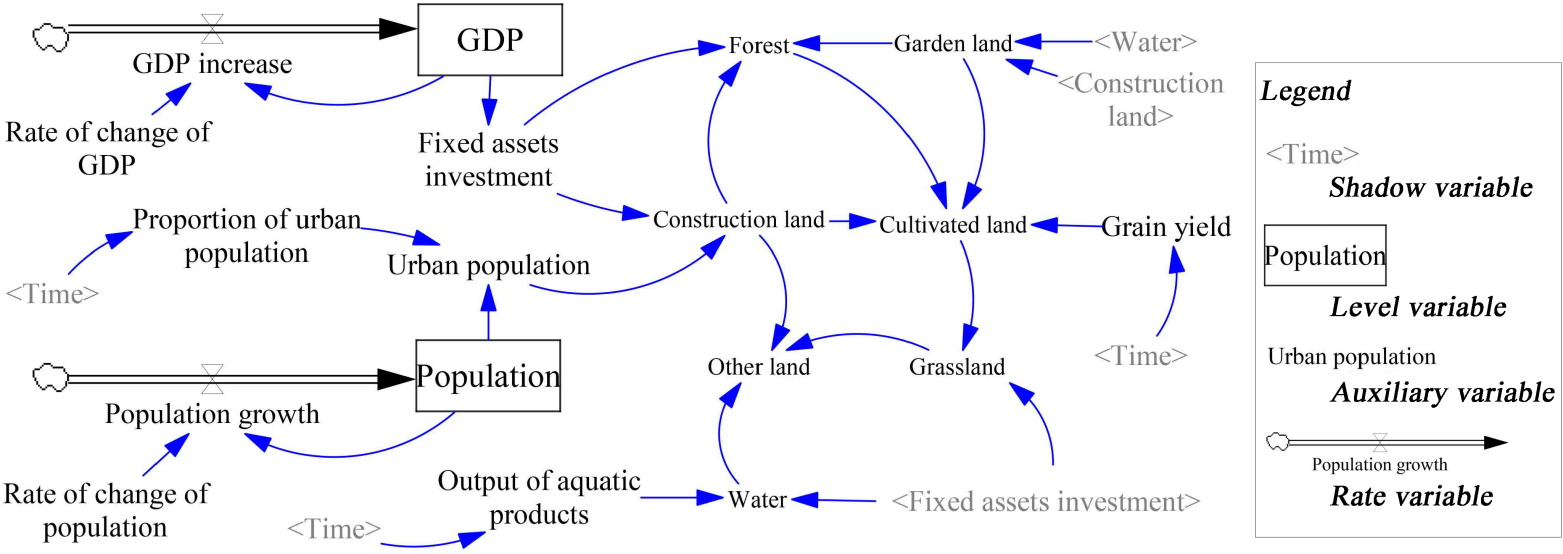
The SD model of land use in Qianshan

Based on the above considerations, the calculation equations for each variable are obtained, with parameters established using techniques such as the arithmetic average method, the trend extrapolation method, and the table function. Finally, we integrated the variable set and the calculating equations to construct the SD model of land use in Qianshan City (Figure 3).

#### 3.1.2 Model verification

The model is debugged and verified based on the existing data of Qianshan city. The model takes 2010 as the basic year, 2011–2018 as the inspection period, and 1 year as the step length. Four indicators including total population, construction land, cultivated land, and forest are selected on a historical inspection basis. The relative error calculation formula is “Relative Error=∣simulated value‐historical value∣/historical value×100%” (Liu, Liang, et al., 2017; Liu, Zheng, et al., 2017; Liu et al., 2020). The simulation results are given in Table 1.

**TABLE 1:** Simulation data and statistic data of land‐use system in Qianshan

Year	2011	2012	2013	2014	2015	2016	2017	2018
Total population								
Historical value/person	587,107	588,358	589,146	583,983	583,662	584,953	585,723	583,969
Simulated value/person	584,262	584,438	584,613	584,788	584,964	585,139	585,315	585,490
Relative error (%)	0.49	0.67	0.77	0.14	0.22	0.03	0.07	0.26
Construction land								
Historical value/km^2^	161.04	159.73	158.94	161.62	161.92	162.37	162.37	162.05
Simulated value/km^2^	158.90	159.52	160.22	161.03	160.46	161.10	161.60	162.36
Relative error (%)	1.33	0.13	0.80	0.37	0.90	0.78	0.47	0.19
Cultivated land								
Historical value/km^2^	437.22	438.13	438.84	436.77	437.56	438.28	438.28	442.64
Simulated value/km^2^	438.49	438.33	438.11	437.81	439.46	439.59	439.95	440.11
Relative error (%)	0.29	0.04	0.17	0.24	0.44	0.30	0.38	0.57
Forest								
Historical value/km^2^	929.30	929.56	930.15	929.12	928.42	927.29	927.29	923.36
Simulated value/km^2^	932.76	932.13	931.39	930.57	930.20	929.38	928.43	927.42
Relative error (%)	0.37	0.28	0.13	0.16	0.19	0.23	0.12	0.44

According to historical test results, the relative errors of the historical and simulated values of the three main state variables: total population, cultivated land, and forest, are all less than 1%, fully complying with the accuracy requirements of the model; the errors of construction land are all less than 2%, also meeting the model's requirements. In this regard, historical tests have proved the efficacy of the land‐use system dynamics model. The model, therefore, can be used to simulate the land‐use situation in Qianshan city from 2019 to 2035.

### 3.2 PLUS model

**FIGURE 4:**
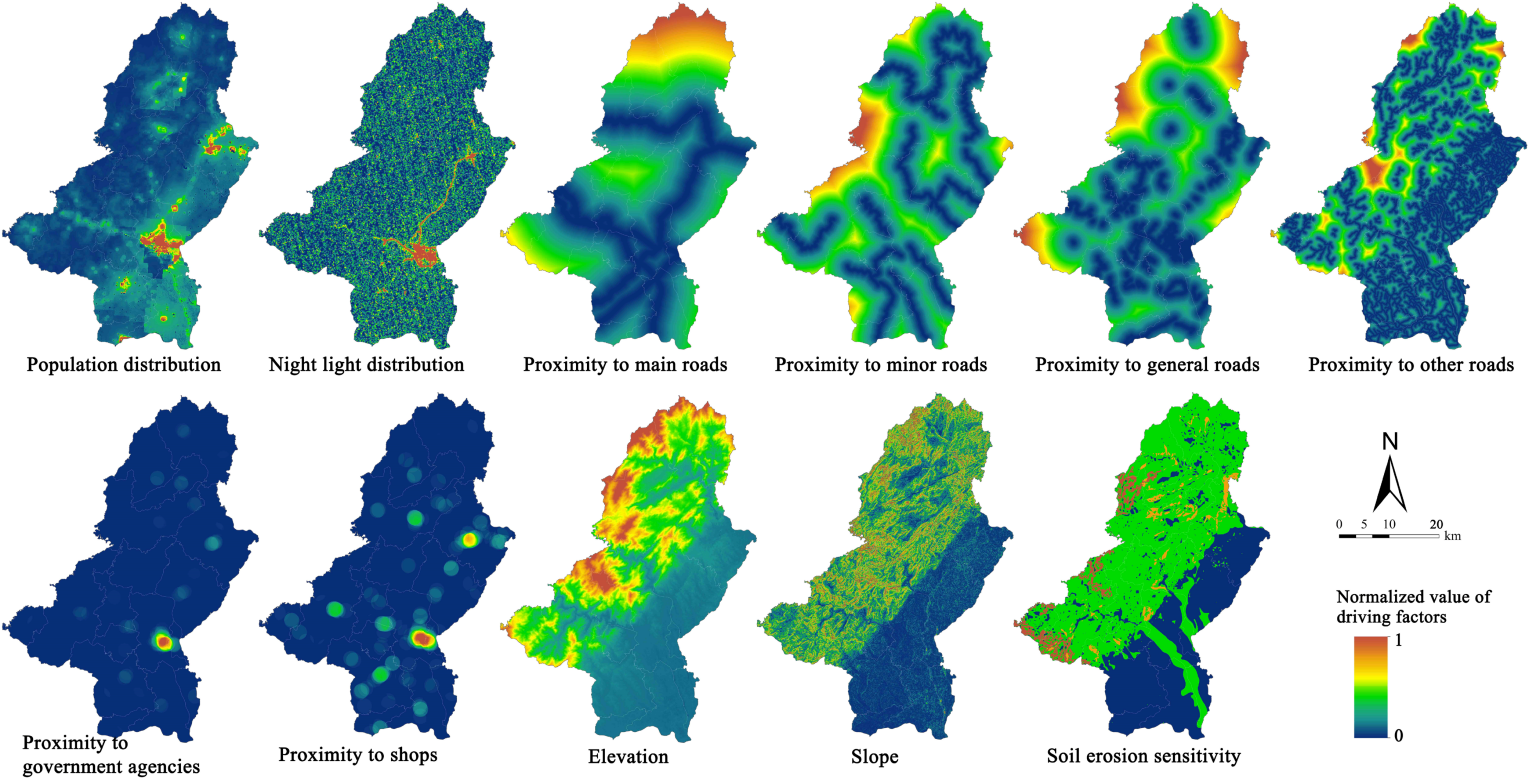
Driving factors which affect the land use in this study

The research uses PLUS model to predict spatial land‐use changes in different scenarios. Socio‐economic data such as roads, population distribution, and night lights, and natural ecological data such as elevation, slope, water, and soil sensitivity are selected as the driving factors of land‐use change (Figure 4). All driving factors were normalized to eliminate the dimensional influence between them and to facilitate direct comparison. In addition, in Figure 2, we mention that the spatial simulation of land‐use change is carried out under the constraints of the restriction map. Specifically, considering that the Qianshan assume important ecosystem functions in the regional context, land‐use changes are regulated by natural and policy factors, so we combined the ecological redline policy (Jiang et al., 2019; Peng et al., 2022) with the permanent basic farmland policy (Zhang & Wu, 2022) to form the restriction map applied in the PLUS model.

The PLUS model, firstly, supported by the Conversion Rule Mining Framework in Land Expansion Analysis Strategy (LEAS), extracts the grids with land‐use changes and randomly selects the sampling points based on two phases of land‐use data. Then the model adopts the random forest algorithm to process the selected points of different land types and thereby generates the conversion rules of the expansion of different land‐use types. Secondly, the PLUS model uses a multi‐type random patch‐generating seed mechanism based on a threshold drop by using the Monte Carlo method. When the neighborhood effect of a certain type of land is equal to 0, the “seed” for change is generated on the probability surface of development of each land‐use type (the output of LEAS). Influenced by the driving factors and conversion rules, the “seed” can generate new land‐use types and grow into new patches formed by a group of cells of the same land‐use type, to achieve the purpose of simulating land‐use changes (Liang et al., 2021).

### 3.3 Evaluation of ecosystem services value

According to the existing studies (Paulin et al., 2020; Sun et al., 2018), the research adopts the equivalent factor method to evaluate the ESV of Qianshan city. Taking into account regional differences, as well as the average grain output of 5968.322 kg/ha and the average grain price of 2.44 yuan/kg in Qianshan from 2003 to 2018, the calculation of the equivalent of an ESV in Qianshan is 2080.356 yuan/ha. The evaluation adopts the table of ESV per unit area proposed by Xie et al. (2015, 2017). In the table, cultivated land equals farmland, forest equals broad‐leaved forest, grassland equals the mean coefficient of scrub‐grassland and meadow, water equals river systems, garden land equals shrubs, and other land equals the mean of desert and bare land. The ESV coefficient per unit area in different land‐use types in Qianshan is then calculated (Table 2).

$$\mathrm{ESV}=\sum_{k=1}^{n}A_{k}\times VC_{k}$$

In the equation, ESV represents the ecosystem services value (yuan); *A*_*k*_ represents the area (ha); VC_*k*_ represents the value coefficient of a certain land‐use type (yuan/ha).

**TABLE 2:** The ESV coefficient per unit area of different land‐use types (yuan/ha)

Ecosystem services	Cultivated land	Grassland	Forest	Water	Garden land	Other land	Construction land
Gas regulation	1851.52	3234.95	4514.37	1601.87	2933.30	135.22	0
Climate regulation	967.37	8560.66	13,522.31	4764.02	8799.91	104.02	0
Water conservation	3110.13	6272.27	9860.89	212,695.60	6969.19	249.64	0
Waste treatment	280.85	2829.28	4015.09	11,545.98	2662.86	426.47	0
Soil formation and protection	1081.79	3942.27	5512.94	1934.73	3578.21	156.03	0
Biodiversity protection	353.66	3588.61	5013.66	5304.91	3266.16	145.62	0
Food production	2298.79	624.11	603.30	1664.28	395.27	10.40	0
Raw material	509.69	925.76	1373.03	478.48	894.55	31.21	0
Entertainment	156.03	1581.07	2205.18	3931.87	1435.45	62.41	0
Total	10,609.82	31,559.00	46,620.78	243,921.74	30,934.89	1321.03	0

## 4 MULTI‐SCENARIO LAND‐USE CHANGE SIMULATION AND ECOSYSTEM SERVICES VALUE RESPONSE

### 4.1 Multi‐scenario land demand analysis

#### 4.1.1 Scenario setting

At present, China's economy is at a “New Normal” stage, and there has been a shift in the growth of the economy from high‐speed to medium‐to‐high‐speed (Zhang et al., 2016). Qianshan city was upgraded from a county into a city in 2018, and its economic base is still relatively weak and its economic volume is small, so the development growth rate of Qianshan will remain higher than the regional average for some time in the future. Under the guidance of relevant literature (Li et al., 2019, 2020; Yu, 2021), combined with the possible future policy directions of Qianshan city, the study selects GDP change rate and population change rate as parameters, and sets three scenarios of high‐growth scenario, medium‐growth scenario, and low‐growth scenario to predict the land‐use changes of Qianshan city from 2019 to 2035, thus exploring the response of ESV.

Considering the Five‐Year Plan is an important tool for guiding social expectations and has a strong guiding effect on economic and social development in China (Hu, 2013; Wang & Gong, 2021). In this study, the adjustment of scenario parameter values is mainly based on the “Fourteenth Five‐Year Plan for National Economic and Social Development of Anhui Province and the Outline of the 2035 Long‐term Goals” and the “Fourteenth Five‐Year Plan for National Economic and Social Development of Qianshan city and the Outline of 2035 Long‐term Goals.” Specifically, the High‐growth Scenario is determined by calculating the maximum GDP growth rate and population growth rate in the last 10 years, except for the outliers. The Medium‐growth Scenario uses the average growth rate of GDP and population of Qianshan city in the past 10 years. And the Low‐growth Scenario is the expected annual average growth rate of Qianshan city in the 14th Five‐Year Plan (and also the actual average growth rate in the 13th Five‐Year Plan). The parameter variables of each scenario are demonstrated in Table 3.

**TABLE 3:** Parameter settings for the different scenarios

Scenarios	GDP change rate (%)	Population change rate (%)	Scenario descriptions
High‐growth rate scenario	13.66	0.52	High population growth; Rapid economic development; Emphasis on economic development while neglecting ecology
Medium‐growth rate scenario	10.40	0.35	Steady population growth, Stable economic development; Economic development is seen as equally important as ecological protection
Low‐growth rate scenario	8.00	0.22	Low population growth; Low economic development; When ecological protection conflicts with economic development, ecological protection will be the first consideration

#### 4.1.2 Simulation results

**FIGURE 5:**
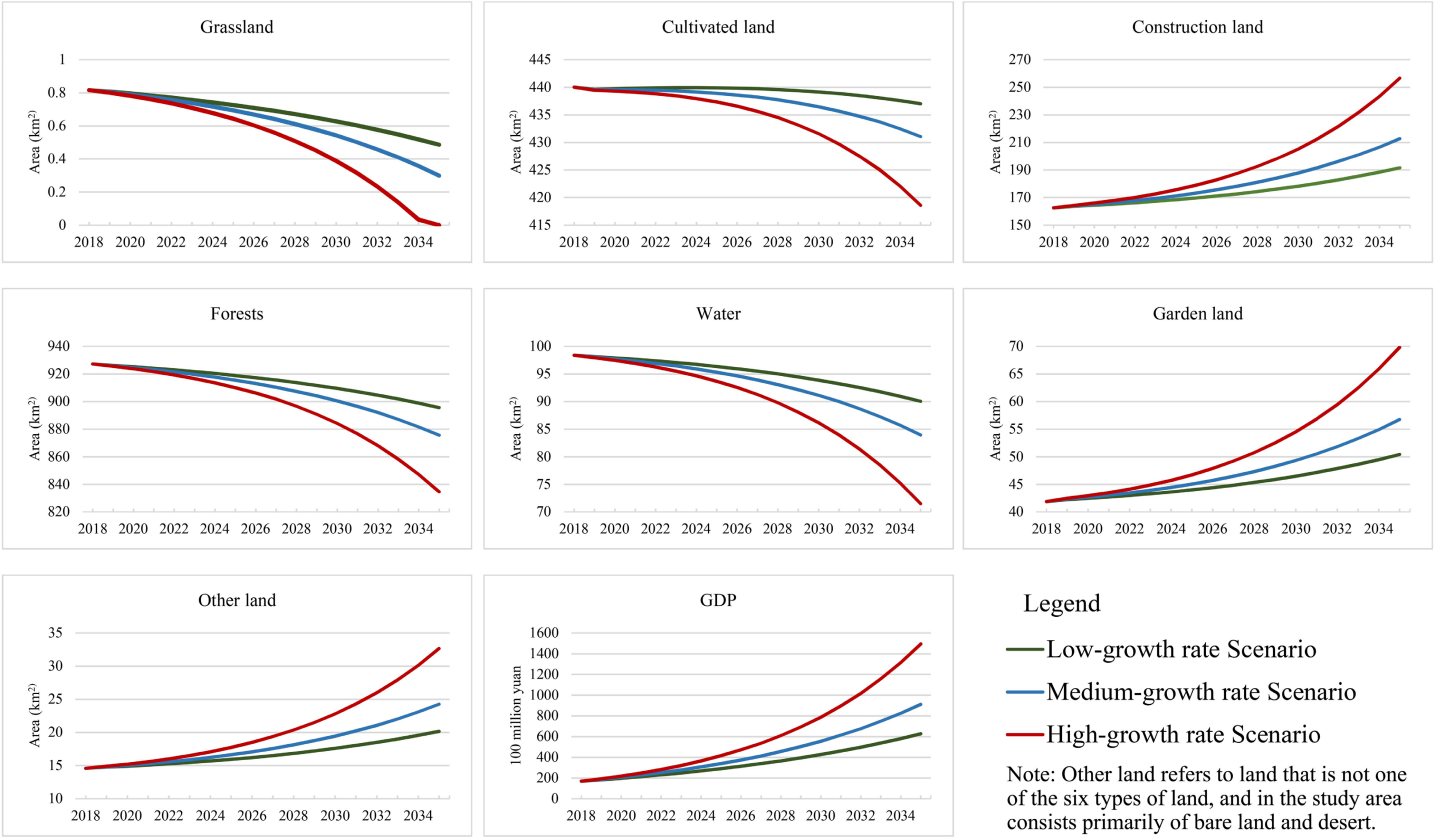
The predicted demand for each type of land use under different scenarios

The Vensim PLE is adopted for the simulation of the three scenarios. The simulation results of land‐use change in Qianshan city in 2035 show (Table 4; Figure 5) that the demand for construction land under different scenarios is increasing, indicating that the expansion of construction land is the prerequisite of economic development. Besides, the other six types of land have roughly the same evolution trend of demand in different scenarios, but the scale of the demand varies greatly. Among them, the demand for grassland has the most significant difference. In the high‐growth rate scenario, grassland will almost be converted to other land. It is related to the development environment and grassland's ecological basis. The rapid development is at the expense of inappropriate reclamation and management, which destroys grassland vegetation and aggravates soil impoverishment. In addition, the grassland ecosystem is relatively less stable compared with forest and water. From the perspective of demand change diversity, cultivated land and forest have a relatively small difference in their demand, indicating that the cultivated land and forest are the major land types that will be invaded for economic development. In the meantime, the garden land for the planting and breeding industry in mountainous cities is also mainly converted from cultivated land and forest.

**TABLE 4:** Simulation results of land use in Qianshan city in 2035

Simulation indexes	Land types/km^2^						
	Grassland	Cultivated land	Construction land	Forest	Water	Garden land	Other land
Low‐growth rate scenario	0.50	437.30	191.58	896.66	90.05	50.44	20.18
Medium‐growth rate scenario	0.32	431.04	212.76	877.62	83.96	56.74	24.26
High‐growth rate scenario	0.01	418.40	256.64	837.64	71.51	69.83	32.68

*Note*: Other land refers to land that is not one of the six types of land, and in the study area consists primarily of bare land and desert.

As the population grows, the demand for food is constantly increasing. If the grain yield cannot be effectively increased, the increase in food demand brought about by population and economic growth will put huge pressure on the demand for cultivated land. According to the high‐growth rate scenario, the most prominent contradiction is between the supply and demand of cultivated land. Among the three modes, the low‐growth rate scenario maintains avoiding too fast economic and population growth. Thus effectively alleviating the pressure on resources and the environment caused by excessive economic growth, while maintaining a stable rate of development also provides the impetus for social sustainability. This mode is also the green development model that China has been promoting in recent years, a way of economic growth and social development that aims at efficiency, harmony, and sustainability (Li et al., 2018).

### 4.2 Multi‐scenario land‐use simulation analysis

#### 4.2.1 Multi‐scenario land‐use change simulation

The land‐use status in 2018 was simulated based on the 2009 land‐use data using the PLUS model, and the results of the simulation were compared with the actual data from 2018. The results indicate that the overall simulation accuracy of the PLUS model was 97.69%, and the kappa coefficient was 0.96. Thus, the accuracy of the model simulation was high, indicating that the model was effective for simulating changes in LULC.

**FIGURE 6:**
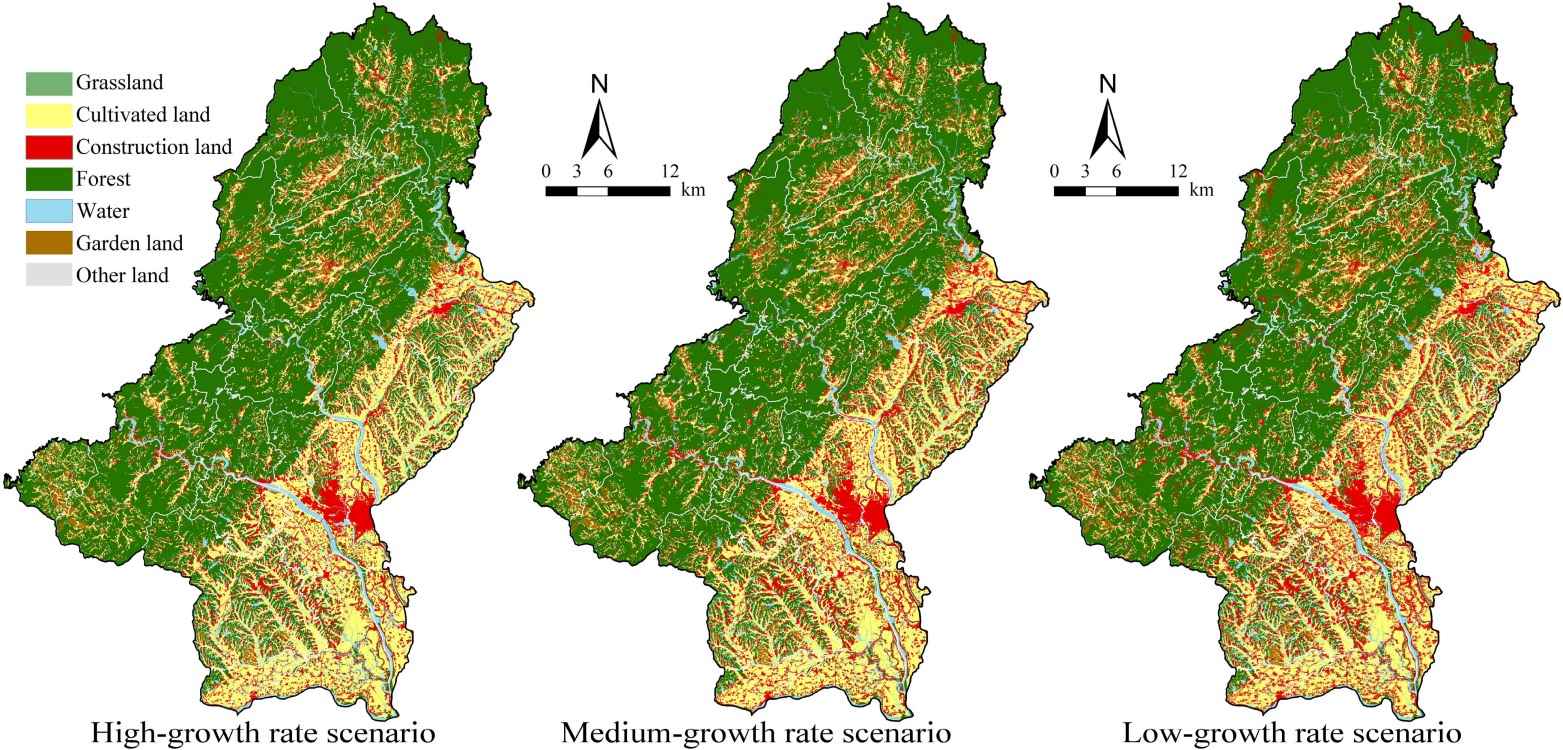
Spatial distribution of LULC under three different scenarios in 2035

The study adopts the PLUS model, based on the land‐use data in 2018, to simulate land‐use changes in three different scenarios. The simulation results in 2035 are shown in Figure 6. Compared with the actual land‐use situation in 2018, the main characteristics of land‐use change in the high‐growth rate scenario are rapid urban expansion and reduction of cultivated land, forest, and water. For the low‐growth rate scenario, it presents the features of a compact distribution of construction land and noticeable achievements in forest and water protection.

**FIGURE 7:**
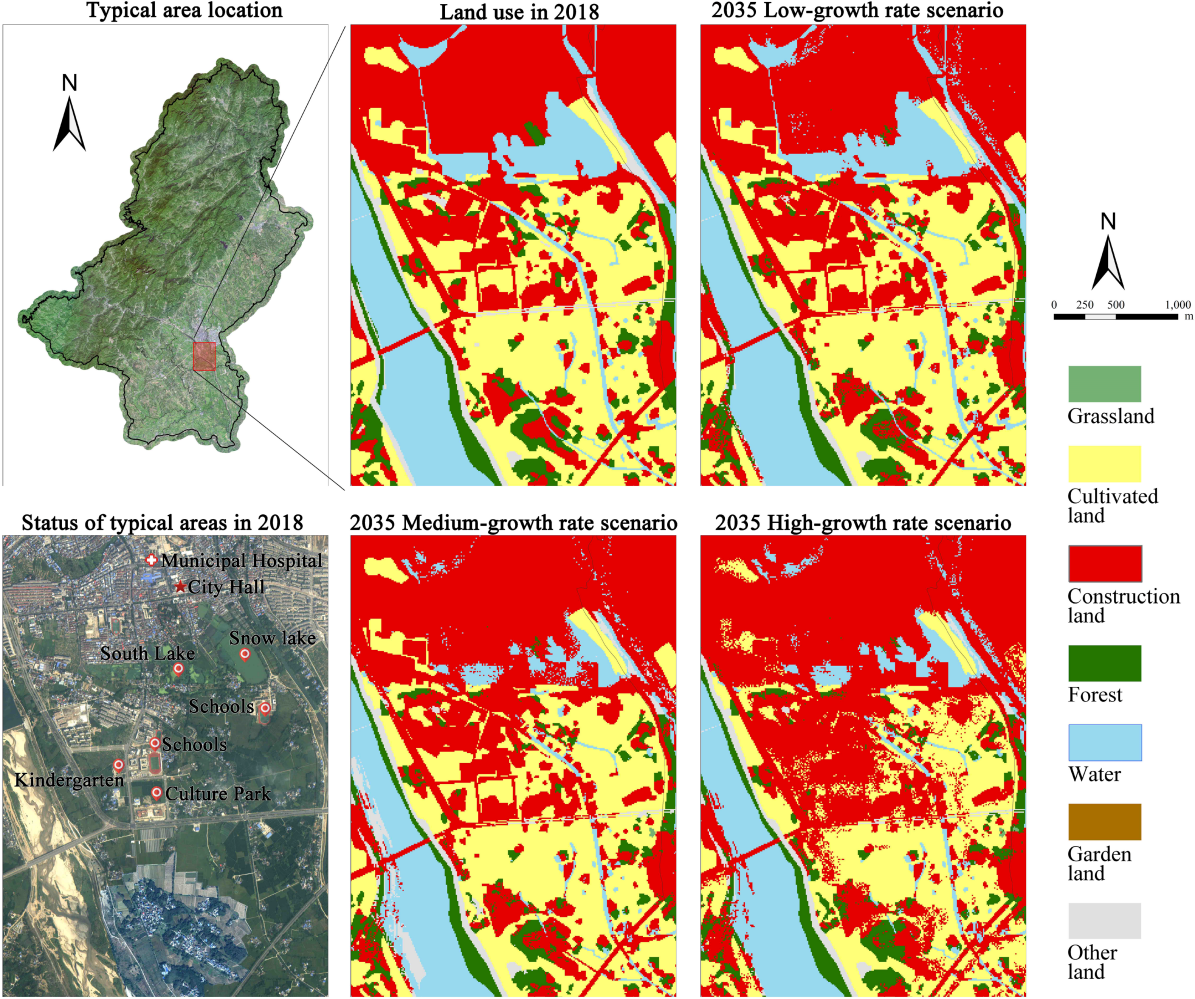
Simulation results of land use in a typical area of Qianshan City in 2035

In the southern area of Qianshan urban area (Figure 7), close to government agencies and central hospitals, several public service facilities such as primary and secondary schools, kindergartens, and parks were built in recent years, indicating that intensive construction activities will be carried out in this area over a certain period. In the scenarios of high‐growth rate and medium‐growth rate, both Snow Lake and South Lake in this area are occupied by construction land on a considerable scale, and a relatively large area of cultivated land is converted to construction land. While in the low‐growth rate scenario, Snow Lake and South Lake generally maintain their status quo, and the expansion of construction land mainly occurs inside the old city, with little expansion outside.

#### 4.2.2 Scenario diversity analysis

**FIGURE 8:**
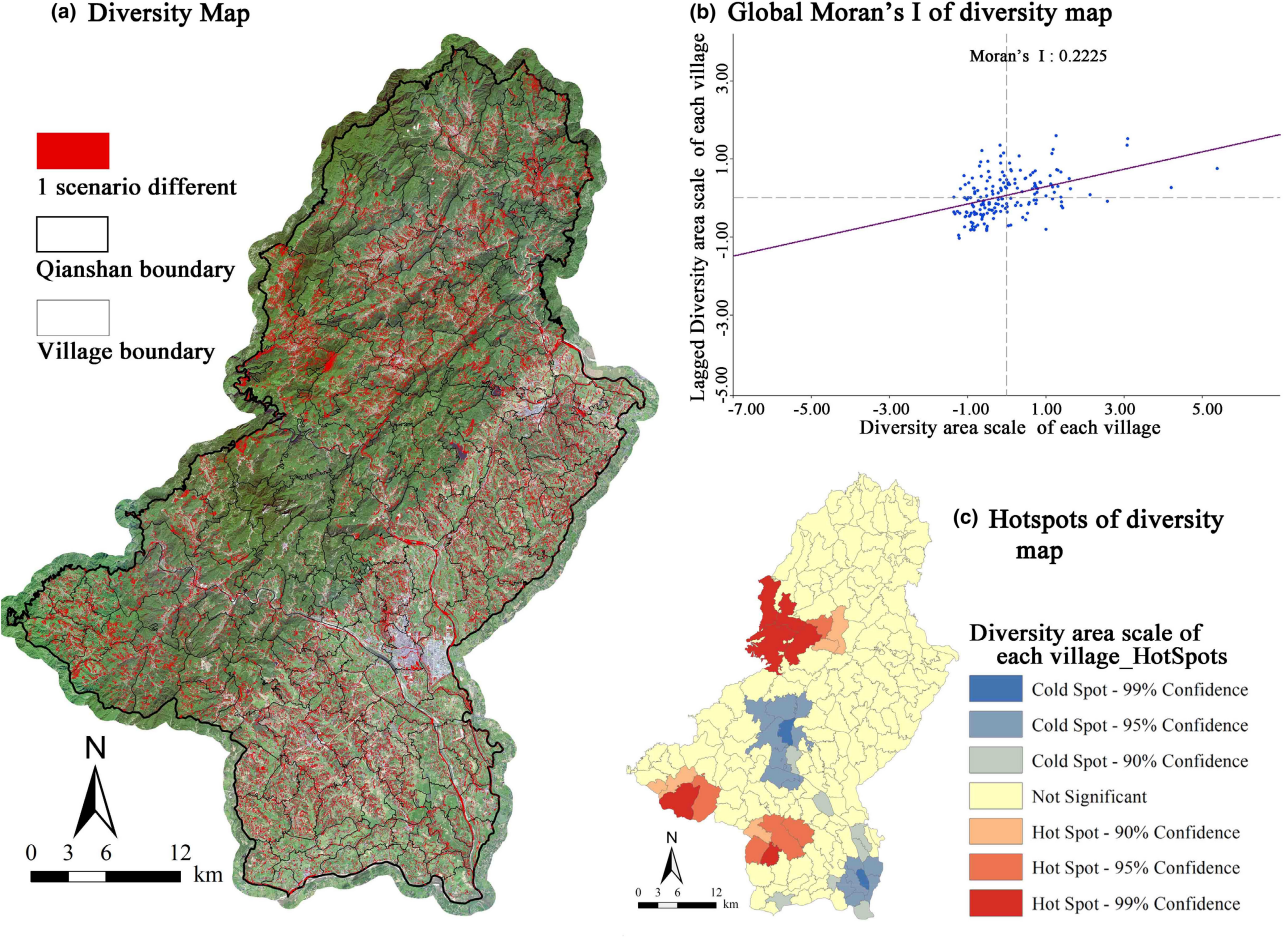
Generating the scenario diversity map in 2035. In subfigure (a), the red patch is the area where the simulation results of each scenario are inconsistent. Subfigure (b) and (c) are the spatial analysis made after counting the total area of various simulation result areas within each village.

There are differences in the simulation of the three scenarios. The red area in Figure 8 demonstrates the areas with diverse results in the three scenarios. We took the village as a spatial unit and calculated the area with diverse result patches within each village. After that, by calculating the global Moran'I (0.2225) of each village's diversity area, it was found that there was a significant spatial autocorrelation in the diversity map of the three scenario simulation results. Therefore, we can calculate the spatial hot and cold spot distribution pattern of the diverse areas of each village. The results show that the differences between the three scenarios are mainly in the western part and along with the southern mountain ranges of Qianshan. In the eastern and central areas, the simulation results are similar for all three scenarios. According to the location of the diversity patches, it is inferred that the main reason for the diversity in the simulation results is the different geographical conditions in each area. The western and northern parts of Qianshan are mountainous areas, with mountainous terrain, steep slopes, and densely distributed water systems, which have multiple development paths. In addition, under the premise of ecological protection, the area may maintain the status quo of forest. However, Qianshan city is rapidly developing its tourism and thereby, spotted construction may appear in this area. In summary, the reason for the diversity of simulation results in different scenarios is not only derived from different scenario settings, but also due to the basic conditions of the land itself.

#### 4.2.3 The driving force of land expansion

**FIGURE 9:**
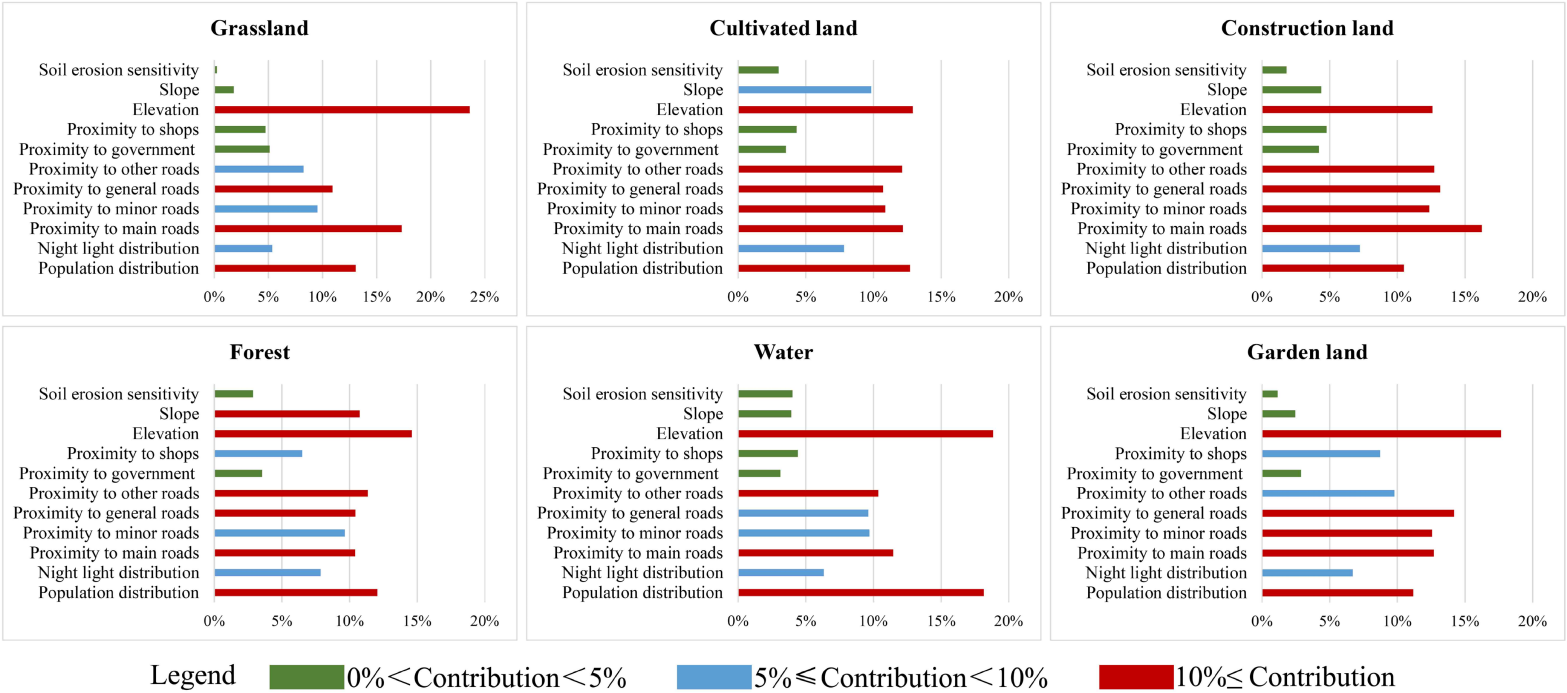
Contribution of each driving factor to the change in land use

The LEAS land‐use change analysis strategy adopted in the PLUS model can effectively obtain the contribution of each driving element to different land‐use changes simultaneously (Figure 9). It is conducive to the exploration of not only the influence of each driving element on land‐use change, but also the understanding of the differences in land‐use simulation results of different scenarios. It is found that elevation has the greatest impact on land‐use changes among all the driving factors. The results show that in areas with land‐use changes and high elevation, other driving factors contribute less, indicating natural activities that influence the land use in the area. The elevation has the greatest impact on grassland, and relatively small impacts on cultivated land and construction land. Roads at all levels have the second most prominent impact on land‐use changes. Among them, construction land is most likely to be affected by roads, and garden area is the least likely. Comprehensively speaking, soil erosion has the least obvious impact on land‐use changes, which indicates that land‐use changes are caused not only by natural geographical conditions but also by human social activities. Among the factors, the distribution of population and night light can most directly reflect the intensity distribution of human activities. After the analysis of the two driving factors, it is found that human activities have the greatest impact on water, followed by forest and cultivated land. The changes are caused by restoring the original land by increasing forest and water and decreasing cultivated land, on the one hand. On the other hand, the conversion to construction land also causes a decrease in the abovementioned land types. It is also indicated that cultivated land or other land can both be likely to be occupied for economic development. Under the premise of preventing soil erosion, it is suggested to moderately develop large tracts of low hilly land, inefficient forest, and abandoned garden land, in order to reduce the occupation of cultivated land for construction.

### 4.3 ESV changes in different scenarios

Significant changes in different land types have caused changes in ESV in the study area. It can be seen from Table 5 that the ESV decreased significantly between 2018 and 2035. The mode of high‐growth rate has caused the greatest decline in ESV, and the ESV declined the least (4.91%) in the low‐growth rate scenario. The results show that an appropriate reduction in the pace of economic development will be conducive to maintaining the stability of ESs.

**TABLE 5:** The ESV change of each ecosystem services type under three different scenarios from 2018 to 2035 (value unit: 100 million yuan)

Ecosystem services type	2009 ESV	2018 ESV	2035 Low‐growth rate scenario			2035 Medium‐growth rate scenario			2035 High‐growth rate scenario		
			ESV	Changes from 2018	Rate of changes from 2018 (%)	ESV	Changes from 2018	Rate of changes from 2018 (%)	ESV	Changes from 2018	Rate of changes from 2018 (%)
Gas regulation	5.32	5.28	5.15	−0.13	−2.41	5.07	−0.22	−4.08	4.88	−0.40	−7.57
Climate regulation	13.90	13.79	13.43	−0.36	−2.59	13.19	−0.60	−4.31	12.69	−1.10	−7.90
Water conservation	32.50	32.17	29.71	−2.46	−7.56	28.26	−3.91	−12.04	25.27	−6.90	−21.25
Waste treatment	5.16	5.12	4.91	−0.21	−4.07	4.78	−0.34	−6.59	4.51	−0.61	−11.83
Soil formation and protection	5.97	5.93	5.78	−0.15	−2.51	5.68	−0.25	−4.21	5.46	−0.46	−7.74
Biodiversity protection	5.51	5.47	5.30	−0.17	−3.08	5.19	−0.28	−5.07	4.96	−0.51	−9.21
Food production	1.76	1.76	1.72	−0.04	−2.46	1.68	−0.08	−4.36	1.61	−0.15	−8.27
Raw material	1.59	1.58	1.54	−0.04	−2.43	1.52	−0.07	−4.10	1.46	−0.12	−7.59
Entertainment	2.59	2.57	2.47	−0.09	−3.54	2.42	−0.15	−5.77	2.30	−0.27	−10.42
Total	74.30	73.66	70.01	−3.65	−4.91	67.77	−5.89	−7.93	63.14	−10.52	−14.16

From the perspective of ESV types, water conservation and waste treatment are reduced the most. The reason might be that a large amount of forest and water is converted into construction land, disappeared due to deforestation, or is used for planting and fish‐farming, resulting in a decrease in the ESV. In addition, the food production value maintains a small and stable reduction, which is mainly due to the most stringent farmland protection policy in China. The increase in garden area also provides certain food production value. However, the study area is located in mountainous areas which have limited food production capacity. In this regard, the future land and space planning should explore the delineation of development and construction changes in Qianshan city of different administrative divisions, reasonably integrate rural settlements, and observe the cultivated land red line to enhance the ESV of cultivated land.

From Table 6, it can be seen that the ESV changes of different land use in Qianshan city from 2018 to 2035 vary in different scenarios. In the high‐growth rate scenario, the ESV of forest accounts for the highest proportion, reaching 61.85%. It is mainly because human activities disrupt a large amount of water and cultivated land, while the forest is well preserved. There is a big gap between the high‐growth rate scenario and actual development due to the excess pursuit of economic growth in the simulated scenario. The ESV of the forest is the highest in all scenarios, with a generally stable proportion of about 60%, though it slowly declines along with development. The proportion of cultivated land in ESV shows an upward trend year‐by‐year. It is because cultivated land remains a relatively stable ESV. In addition, it also results from the overall ESV decline in the study area. The development of the characteristic planting industry in Qianshan city expands the garden area, as well as its ESV. The simulated results of the medium‐growth rate mode show an increase in the ESV of garden land from 138 million yuan in 2018 to 176 million yuan in 2035. Generally, the ESV of grassland decreases the most, and cultivated land and forest exhibit a relatively stable trend in their ESV, while the ESV of garden land demonstrates an upward trend year‐by‐year.

**TABLE 6:** The ESV change of each land‐use type under three different scenarios from 2018 to 2035 (value unit: 100 million yuan)

Land‐use type	2009 ESV	2018 ESV	2035 Low‐growth rate scenario			2035 Medium‐growth rate scenario			2035 High‐growth rate n scenario		
			ESV	Changes from 2018	Rate of changes from 2018 (%)	ESV	Changes from 2018	Rate of changes from 2018 (%)	ESV	Changes from 2018	Rate of changes from 2018 (%)
Cultivated land	4.68	4.70	4.64	−0.06	−1.21	4.57	−0.12	−2.63	4.44	−0.26	−5.50
Grassland	0.03	0.03	0.02	−0.01	−44.10	0.01	−0.02	−61.66	0.00	−0.03	−91.69
Forest	43.49	43.05	41.80	−1.24	−2.86	40.92	−2.13	−4.90	39.05	−4.00	−9.19
Water	24.81	24.51	21.96	−2.55	−10.27	20.48	−4.03	−16.25	17.44	−7.07	−28.50
Garden land	1.28	1.35	1.56	0.21	16.11	1.76	0.40	31.31	2.16	0.81	62.93
Other land	0.02	0.02	0.03	0.01	53.83	0.03	0.01	86.46	0.04	0.03	153.81
Total	74.30	73.66	70.01	−3.65	−4.91	67.77	−5.89	−7.93	63.14	−10.52	−14.16

**FIGURE 10:**
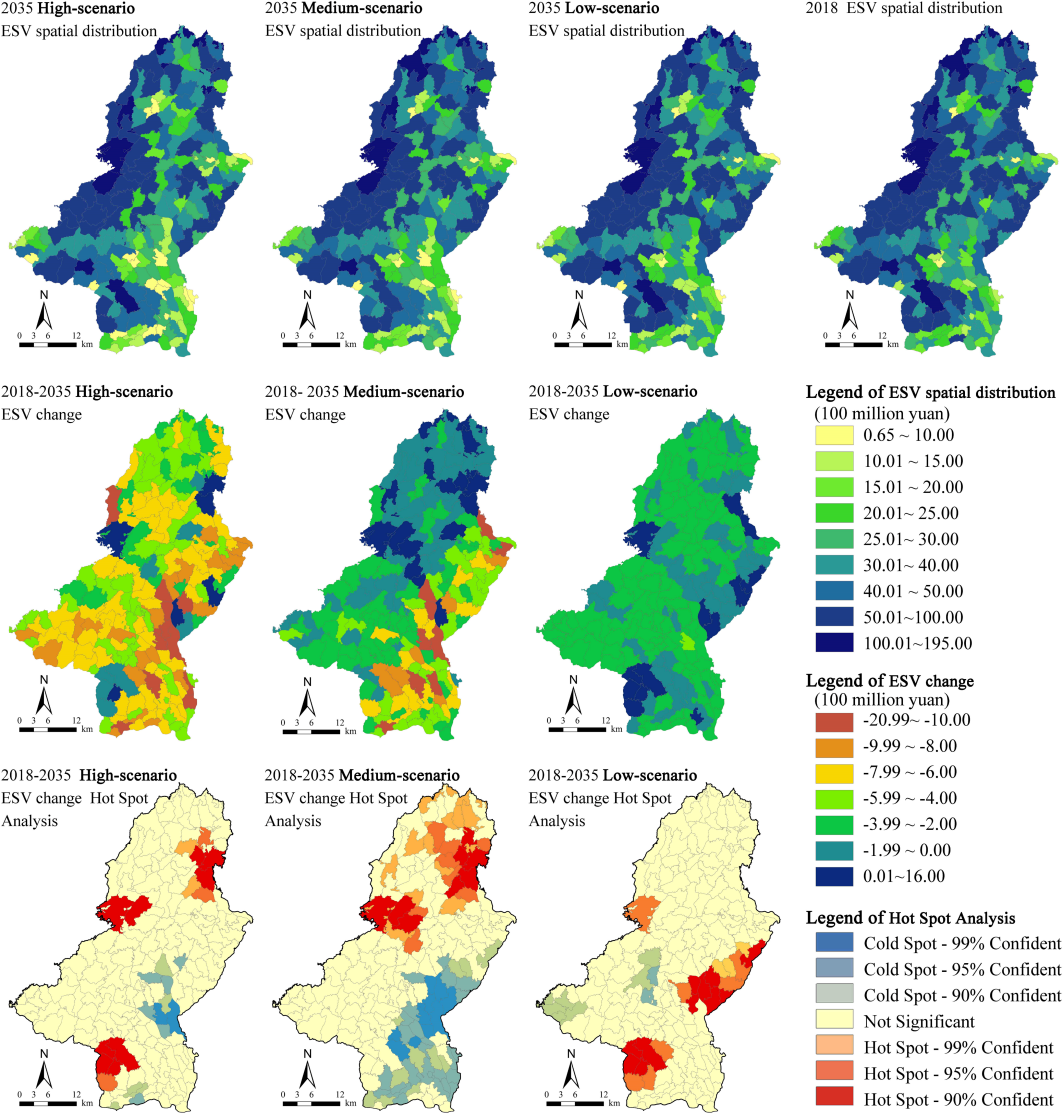
Spatial distribution of ESV from 2018 to 2035

The study takes the village‐level units of Qianshan as the geographical boundary to calculate the ESV of each village and displays it in Figure 10. The ESV is the highest along the western mountainous area, and relatively low in the urban construction area of Qianshan city. Urban development and construction land expansion cause small variations in the spatial distribution of ESV, indicating that areas in the study area develop relatively balanced. However, the ESV changes show a certain difference in space of each village. In the high‐growth rate and medium‐growth rate scenarios, the ESV falls most significantly around the city center, while it increases significantly in the central and northeastern regions. In the low‐growth rate scenario, the ESV of the downtown area somewhat increases, reflecting the development idea of urban construction concessions to ecological protection. In the medium‐growth rate scenario, the ESV of each village has a small change and exhibits a relatively stable trend of development.

## 5 DISCUSSION

The simulation results of this study are identical to those of related studies on land‐use simulations at different scales in Anhui Province (Hu et al., 2020), Yangtze River Delta (Zhang, Wang, et al., 2020; Zhang, Zhou, & Song, 2020), where Qianshan is located. In this study, the changes in land use and ESV under three different scenarios demonstrated significant spatial heterogeneity. The study revealing that the expansion trend of construction land is evident, while the size of forest and water is rapidly decreasing. The expansion of built‐up areas can squeeze the forest and reduce the supply of ESs from the forest. This can lead to a drop in ESV (Delphin et al., 2016), especially in the areas near built‐up areas, which suggests that land‐use change is the main cause of the drop in ESV (Jiang et al., 2017).

Subsequently, the spatial simulation results showed that the construction land was expanded on a large scale in the built‐up areas, while in other areas it was a decentralized dot‐like expansion, and that there was a significant spatial autocorrelation in the transformation of various types of land use, indicating that the change was not random. Large‐scale (Zhang, Wang, et al., 2020; Zhang, Zhou, & Song, 2020) and small‐scale (Peng et al., 2022) studies in other parts of China have confirmed this change in land use over time.

In the study, the topographic factors (elevation), followed by population distribution and traffic conditions, had the most impact on land‐use conversion in the small‐scale range of mountainous areas. This is different from the main demographic and economic effects of land change in China's coastal areas (Du et al., 2014) and the western region (Yang et al., 2022). It is indicating that land‐use conversion is primarily the result of human transformation activities based on natural topographic conditions.

Finally, we calculated the future ESVs under various scenarios in the study area, with the ESV changes in forest and water being the most significant from the standpoint of various land‐use types. From the perspective of various specific ESVs, the decline in the study area's water conservation capacity is the most apparent. As with other findings, ESV changes were most influenced by land‐use patterns in forest and water (Aziz, 2021; Rahman & Szabó, 2021).

During model construction, the 2018 simulation results were compared with the actual situation, and the total amount of land used in SD simulation and the spatial distribution of various types of land use simulated by PLUS showed high accuracy, indicating that the SD‐PLUS model constructed in this study can accurately simulate the land‐use change in mountainous areas at the county scale.

There are three major distinctions between the small‐scale mountain areas land‐use simulation examined and big cities in this study. First, the land‐use affecting factors and drivers identified by this study are distinct from those of big cities. The study used population, economics, fixed asset investment, and the interaction between different types of land use as the primary variables in the SD model because the effect of population and economic factors on land‐use change is more pronounced in small cities than in large cities. Simultaneously, the composition of natural ecosystems in mountainous regions is more complex than in other landscape types, and land‐use changes are more spatially sensitive. Consequently, when using PLUS for spatial simulation, the study selected natural environmental data (e.g. elevation, slope), socioeconomic data, and demographic data (e.g. population distribution, night light distribution). Secondly, in contrast to big cities, this case is a mountainous location with critical ESs in the region. Therefore, this study uses the ecological redline and permanent basic farmland redline as the restrictive maps, in conjunction with China's current cultivated land and ecological protection policies, so that the land use within this range does not change, thus more accurately reflecting the actual situation. Third, the land‐use simulation model applied to big cities lacks a flexible mechanism for dealing with changes in multiple types of land‐use patches and cannot simulate land‐use changes at fine scales. So, this study uses the PLUS model, which takes into account the statistical relationships between spatial land‐use allocation and driving factors as well as the competitive relationships between different land‐use approaches (Liang et al., 2021).

Land‐use change is considered to be the greatest threat to nature, leading to a decline in the abundance, diversity, and health of species and ecosystems globally (Davison et al., 2021). Land‐use change can have a direct impact on species through habitat destruction and environmental modification (Bender et al., 1998). The simulation of land use and ESV in small‐scale mountainous areas in this study can help to reconcile land‐use conflicts and is essential for adjusting regional policies on population, industry, and environmental protection and for reconciling human–land relationships, which is important for the precise implementation of biodiversity conservation strategies (Zhao et al., 2015) and ecosystem management decisions (Fu et al., 2019).

Qianshan city is experiencing rapid urbanization. At this stage, land resources are typically utilized for production and living purposes, such as houses, factories, transportation, and parks. The city's original green land and water are also at risk of being encroached upon. Consequently, optimizing land use structures and protecting forests and water to increase the regional ecosystem service value is a critical work for local governments. On the one hand, the economic development rate should be appropriately slowed down, and the transformation of economic development from "high speed" to "high quality" should be encouraged. On the other hand, through afforestation, land that is unsuitable for construction and breeding can be converted into forest to maintain the stability of forest ecosystems and expand the area of forest, thereby enhancing the capacity of regional ESs. The model constructed in this study simulates land‐use changes in various scenarios, visually displaying areas that may be occupied and assisting in delimiting urban construction prohibited areas. The simulation results also indicate that the expansion of construction land is relatively dispersed. Therefore, in the current territorial spatial planning work being conducted by Chinese governments at all levels, we should make full use of the unused land between the construction land, perform well in stock excavation, and to conserve land resources and enhance land‐use efficiency.

In this study, the ESV changes under different scenarios are intuitively reflected through scenario simulation using the PLUS model. Data collection is relatively straightforward, and the research results are reliable. However, several influential factors, such as decision‐making of policies, social orientation, and human factors, are not included in the study. The later research should further understand the mechanisms of complex urban system evolution under the influence of human factors, refine land‐use types, and improve the accuracy and feasibility of research results.

## 6 CONCLUSION

The study takes the Qianshan city in Anhui province as the research object, based on current land use, to predict the changes in demand for land use and spatial response in the study area by the SD‐PLUS model. In addition, it also discusses the temporal and spatial evolution of ESV. The main conclusions are as follows:

1. It is predicted that the scale of construction land in Qianshan city by 2035 will reach at least 191 km^2^ (in the low‐growth rate scenario). And the garden land has increased obviously along with the development of characteristic planting industry, while the scale of forest and water exhibit the most significant decrease.
2. It is found that construction land expands in blocks in the built‐up areas of cities and towns and expands in spots in mountainous areas. The land‐use changes in multiple scenarios are spatially different and demonstrate spatial autocorrelation. It is mainly because the western and southern parts of the study area are mountainous areas with mountainous terrain, steep slopes, and dense distribution of water systems. In this regard, the study area has various possibilities for change in different social contexts. According to the analysis of the driving factors of land‐use change, elevation has the most significant impact on the conversion of each type of land, followed by two major factors: road facilities and population distribution.
3. The ESV declines significantly from 2018 to 2035, and the low‐growth rate scenario drops the least (4.91%). From the ESV types, water conservation and waste treatment decrease the most in value, while food production exhibits a relatively small and stable trend of value decrease. From the perspective of land types, the ESV of grassland decreases the most, and cultivated land and forest exhibit a relatively stable trend in their ESV, while the ESV of garden land demonstrates an upward trend year‐by‐year. At the spatial level, the ESV is the highest in the western mountainous area, and relatively low in the urban construction area.

## AUTHOR CONTRIBUTIONS

Yao Li, Jiulin Li and Jinlong Chu conceived and designed the experiments. Yao Li performed the experiments. Yao Li, Jiulin Li and Jinlong Chu analyzed the data and wrote the manuscript.

## ACKNOWLEDGEMENTS

The authors appreciate the support provided by the Anhui Province Philosophy and Social Science Planning Youth Project (Grant no. AHSKQ2021D77), National Key R&D Program of China (Grant no. 2017YFC0702503), and National Natural Science Foundation of China (Grant no. 51678001).

## CONFLICT OF INTEREST

The authors declare that there is no conflict of interest regarding the publication of this paper.

## Data Availability

Data to reproduce results are available in an open archive hosted by the Open Science Framework (https://doi.org/10.17605/OSF.IO/R4SZU).
